# Perineural Mast Cells Are Specifically Enriched in Pancreatic Neuritis and Neuropathic Pain in Pancreatic Cancer and Chronic Pancreatitis

**DOI:** 10.1371/journal.pone.0060529

**Published:** 2013-03-28

**Authors:** Ihsan Ekin Demir, Stephan Schorn, Elisabeth Schremmer-Danninger, Kun Wang, Timo Kehl, Nathalia A. Giese, Hana Algül, Helmut Friess, Güralp O. Ceyhan

**Affiliations:** 1 Department of Surgery, Klinikum rechts der Isar, Technische Universität München, Munich, Germany; 2 Key laboratory of Carcinogenesis and Translational Research (Ministry of Education), Department of Hepatic, Biliary and Pancreatic Surgery, Peking University School of Oncology, Beijing Cancer Hospital and Institute, Beijing, China; 3 Department of Internal Medicine II, Klinikum rechts der Isar, Technische Universität München, Munich, Germany; 4 Department of General Surgery, University of Heidelberg, Heidelberg, Germany; The Chinese University of Hong Kong, Hong Kong

## Abstract

**Background:**

Pancreatic neuritis is a histopathological hallmark of pancreatic neuropathy and correlates to abdominal neuropathic pain sensation in pancreatic adenocarcinoma (PCa) and chronic pancreatitis (CP). However, inflammatory cell subtypes that compose pancreatic neuritis and their correlation to the neuropathic pain syndrome in PCa and CP are yet unknown.

**Methods:**

Inflammatory cells within pancreatic neuritis lesions of patients with PCa (n = 20) and CP (n = 20) were immunolabeled and colorimetrically quantified with the pan-leukocyte marker CD45, with CD68 (macrophages), CD8 (cytotoxic T-lymphocytes), CD4 (T-helper cells), CD20 (B-lymphocytes), NCL-PC (plasma cells), neutrophil elastase, PRG2 (eosinophils), anti-mast cell (MC) tryptase and correlated to pain sensation. Perineural mast cell subtypes were analyzed by double immunolabeling with MC chymase. Expression and neural immunoreactivity of protease-activated receptor type 1 (PAR-1) and type 2 (PAR-2) were analyzed in PCa and CP and correlated to pain status of the patients.

**Results:**

In PCa and CP, nerves were predominantly infiltrated by cytotoxic T-lymphocytes (PCa: 35% of all perineural inflammatory cells, CP: 33%), macrophages (PCa: 39%, CP: 33%) and MC (PCa: 21%, CP: 27%). In both entities, neuropathic pain sensation was associated with a specific increase of perineural MC (PCa without pain: 14% vs. PCa with pain: 31%; CP without pain: 19% vs. CP with pain: 34%), not affecting the frequency of other inflammatory cell subtypes. The vast majority of these MC contained MC chymase. PAR-1 and PAR-2 expression did not correlate to the pain sensation of PCa and CP patients.

**Conclusion:**

Pancreatic neuritis in PC and CP is composed of cytotoxic T-lymphocytes, macrophages and MC. The specific enrichment of MC around intrapancreatic nerves in neuropathic pain due to PCa and CP suggests the presence of MC-induced visceral hypersensitivity in the pancreas. Therefore, pancreatic and enteric neuropathies seem to share a similar type of neuro-immune interaction in the generation of visceral pain.

## Introduction

Inflammation and cancer are intertwined in the generation, course and outcome of human malignancies. A specific and unique subtype of cancer-related inflammation is encountered around nerves in pancreatic tumours, especially in pancreatic cancer (PCa) and in the inflammatory pancreatic head tumour associated with chronic pancreatitis (CP). Indeed, both of these tumours frequently contain focal inflammatory cell clusters around intrapancreatic nerves [1], [2]. In his seminal electron-microscopic study on nerves in CP, Dale Bockman reported on the presence of severe damage in such nerves which were specifically infiltrated by inflammatory cells [3]. Later studies made the deciding contribution related to the importance of this targeted neural immune cell infiltration termed *pancreatic neuritis* in PCa and CP patients: Increasing frequency and severity of pancreatic neuritis have been shown to bear a major correlation to the severity of abdominal pain sensation and neuroplastic alterations in PCa and CP patients [1], [4], [5].

Mechanisms of pancreatic neuritis remain to be elucidated. Regarding the inflammatory mediators involved in pancreatic neuritis, interleukin-8 (IL-8), the neuronal chemokine fractalkine and its receptor CX3CR1 have been shown to be overexpressed in nerves in CP tissue, and increased endoneural fractalkine presence was detected to correlate to the severity of pancreatic neuritis, tissue macrophage infiltration and pain sensation [6]–[8].

The exact subtypes and characteristics of the immune cells infiltrating pancreatic nerves are yet unknown. In the only study related to this question, Keith et al. demonstrated in a semi-quantitative fashion the increased presence of eosinophils around nerves in CP and the association between pain sensation and the extent of perineural eosinophilic infiltration [9].

A better understanding of characteristics of the perineural inflammatory cell infiltrate in PCa and CP is likely to allow a deep insight into the mechanisms of pancreatic neuritis. Therefore, in the present study, we aimed at providing a systematic quantitative characterization of pancreatic neuritis-associated inflammatory cell clusters in PCa and CP. For this purpose, we quantified peri- and endoneural leukocytes in normal human pancreas (NP), PCa and CP. Furthermore, we investigated the quantitative distribution of a large panel of leukocyte subset markers in PCa and CP tissue, including CD68 (macrophages), CD8 (cytotoxic T-lymphocytes), CD4 (T-helper cells), CD20 (B-lymphocytes), NCL-PC (plasma cells), neutrophil elastase, proteogylcan 2 / PRG2 (eosinophils) and anti-mast cell (MC) tryptase and chymase within neural inflammatory clusters. Finally, we correlated the amount of these neural inflammatory cell subsets, and the expression of two potential receptors (protease-activated-receptor/PAR-1 and PAR-2) for MC-derived proteases to the neuropathic pain sensation of PCa and CP patients.

## Materials and Methods

### Ethics statement

The study was approved by the ethics committees of the Technische Universität München, Munich, Germany and the University of Heidelberg, Germany.

### Patients and tissues

Pancreatic tissue samples for immunohistochemistry were collected from patients following pancreatic head resection for pancreatic cancer (PCa, n  =  20; male/female  =  8/12, median age  =  66 years) and chronic pancreatitis (CP, n  = 20; male/female  =  13/7, median age  =  51 years). All patients were informed, and written consent was obtained for tissue collection. According to the international classification of the UICC (2009), all patients had stage IIb pancreatic cancer. The etiology of CP was alcoholic in all patients. Due to frequently observed concomitant inflammatory process at the resection margins of pancreatic tissue specimens, normal pancreatic tissue samples were obtained from healthy organ donors (NP, n  =  10; male/female  =  6/4, median age  =  38 years) whenever there was no suitable recipient for transplantation available. The tissue collection was approved by the ethics committees of the Technische Universität München, Munich and University of Heidelberg, Germany. The resected pancreatic tissue samples were divided into parts which were immediately fixed in 4% paraformaldehyde followed by paraffin-embedding, as described previously [1], [10].

### Abdominal pain

In all PCa and CP patients, the individual pain status (Pain vs. No Pain) and the individual pain score (pain intensity and frequency) were prospectively registered prior to the operation, as described previously [2]. Pain intensity was graded by using a short scale: 0  =  none, 1 =  mild, 2  =  moderate and 3  =  strong pain. Pain frequency was graded as 3  =  daily, 2  =  weekly and 1  =  monthly. To calculate the severity of pain, pain intensity and pain frequency of each individual were multiplied. According to the final pain score, the patients were divided into three subgroups: Pain I (0) representing the group of patients without pain, Pain II (1–3) patients who suffered from mild pain and Pain III (4–9), with moderate to severe pain.

### Immunohistochemistry & double immunofluorescence labeling

Consecutive 3 µm sections from paraffin-embedded NP, PCa and CP samples were analyzed for the pan-neuronal marker Protein Gene Product 9.5 (PGP 9.5) and for inflammatory cell surface markers including cluster of differentiation 45 (CD45) as pan-leukocyte marker, CD8 as marker of cytotoxic T lymphocytes, CD4 to label T helper lymphocytes, CD68 as marker of macrophages, neutrophil elastase (NE) for neutrophilic granulocytes, CD20 for B-lymphocytes, NCL-PC for plasma cells, PRG2 for eosinophils and anti-mast cell (MC) tryptase and anti-MC chymase to identify mast cells. The protease-activated receptor type 1 (PAR-1) and type 2 (PAR-2) were immunolabeled in additional consecutive sections. The sections were incubated with the primary antibodies at the indicated dilutions overnight in a humid chamber at 4°C (Table 1). The negative controls were incubated with the same IgG or IgM subclass non-immunized antibodies. Primary antibody dilutions were performed with normal goat serum. All antigens were detected through the DAKO Envision System-HRP (Hamburg, Germany) or by Alexa Fluor^®^ 488 and 594 antibodies (Invitrogen, Germany) for the matching species. DAB was used as chromogen. Digital imaging was performed with the Keyence Biorevo BZ-9000 system (Keyence, Neu-Isenburg, Germany).

**Table 1:** Primary antibodies.

Antibody	Species	Type	Dilution	Source
Anti - PGP 9.5	Mouse	Monoclonal	1∶ 1000	DAKO, Hamburg, Germany
Anti – CD45	Rabbit	Polyclonal	1∶ 500	Antibodies Online, Aachen, Germany
Anti – CD8	Rabbit	Polyclonal	1∶ 60	Diagnostic BioSystems, CA, USA
Anti – CD4	Rabbit	Polyclonal	1∶ 60	Monosan, Uden, Netherlands
Anti – CD68	Mouse	Monoclonal	1∶ 60	Diagnostic BioSystems, CA, USA
Anti-neutrophil elastase (NE)	Rabbit	Polyclonal	1∶ 2000	Abcam, Cambridge, UK
Anti-CD20	Mouse	Monoclonal	1∶500	Novocastra/Leica, Wetzlar, Germany
Anti-PRG2	Rabbit	Polyclonal	1∶500	Sigma-Aldrich, Munich, Germany
Anti-NCL-PC	Mouse	Monoclonal	1∶16000	Novocastra/Leica, Wetzlar, Germany
Anti-Mast cell tryptase	Rabbit	Monoclonal	1∶800	Abcam, Cambridge, UK
Anti-Mast-cell-chymase	Goat	Polyclonal	1∶200	Acris Antibodies, Herford, Germany
Anti-PAR-1	Mouse	monoclonal	1∶100	Santa Cruz Biotech., Germany
Anti-PAR-2	Rabbit	Polyclonal	1∶4000	LifeSpan/Biozol, Eching, Germany

### Quantitative analysis of inflammatory cell distribution around nerves

In order to determine the amount of inflammatory cell subtypes around intrapancreatic nerves, the immunostained area occupied by each cell subtype within neural inflammatory cell clusters was measured via the ImageJ software (ImageJ 1.36b, Wayne Rasband). For this purpose, from each section of a patient, between three to five nerves demonstrating pancreatic neuritis were first identified and subsequently photographed with the help of a consecutive PGP9.5-stained section and a consecutive hematoxylin-eosin-stained section. Pancreatic neuritis was histologically defined and identified as “a cluster of neural inflammatory cells which is in contact with the perineurium and/or endoneurium and can clearly be delineated from the remaining general tissue inflammatory cell infiltrate”. After conversion of the RGB image to an 8-bit image, threshold function was used to define a phase with the help of a pre-set threshold which solely labels the area occupied by the immunostained neural inflammatory cells, and the software automatically determined the absolute and per cent area of this phase on each image, as also described previously [10]. The absolute areas measured on each image for the immunoreactivity of CD8, CD4, CD68, CD20, NCL-PC, PRG2, NE, anti-MC-tryptase and -chymase were then related to that for CD45 as the pan-leukocyte marker to determine the distribution of inflammatory cell subsets in the pancreatic neuritis cell population. The photographing and subsequent quantitative analysis were performed by three observers (SS, IED and ESD) blinded to clinical and immunohistochemical data, as described previously [1].

### Real-time Light Cycler® Quantitative-Polymerase-Chain-Reaction (QRT-PCR)

Extraction of mRNA from pancreatic tissue was prepared by using TissueLyser II and the RNeasy plus kit (Qiagen/Hilden) according to the manufacturer's instructions. Subsequently, RNA quantity and purity was determined using Nanodrop ND1000 (Peqlab/Erlangen). First-strand cDNA synthesis was performed with the Transcriptor-First-Strand cDNA Synthesis kit (Roche/Mannheim) according to the manufacturer's instructions. Expression of protease-activated-receptor type 1 (PAR-1, GeneBank, GeneID:2149), protease-activated-receptor type 2 (PAR-2, GeneBank, GeneID:2150) and of the reference housekeeping gene cyclophilin-B (CypB, GeneBank, GeneID:5479) were measured with the Roche LightCycler-480 Real-Time PCR System and LightCycler-480 SYBR Green I Master kit. In accordance with the Pfaffl method [11], relative expression was based on the mean crossing-point deviation between the 3 samples normalized to the mean crossing-point deviation for the reference gene, after efficiency correction of the PCR reactions. The relative expression of PAR-1 and PAR-2 in samples was then normalized to the level in normal human pancreas (NP). All primers were obtained from Sigma-Aldrich.

### Quantification of neural immunoreactivity for PAR-1 and PAR-2

On each section, the mean neural immunoreactivity for PAR-1 and PAR-2 was determined via ImageJ-based colorimetry, as also shown previously [10]. Briefly, immunolabeled nerves with pancreatic neuritis from each section with PCa or CP were photomicrographed and subjected to the threshold function of the software after conversion into an 8-bit image. The per cent immunostained area in each nerve was determined by the software after setting a defined threshold in nerves as regions of interest for each staining (i.e. PAR-1 and PAR-2) and corresponded to neural immunoreactivity. The mean immunoreactivity of each patient was calculated by determining the average neural immunoreactivitiy of all nerves with pancreatic neuritis in each patient.

### Statistical analysis

Statistical analysis was performed using the GraphPad Prism 5 Software (La Jolla, CA, USA). For multiple comparisons and due to the interdependence of the measured cell subset areas and percentages within a given neuritis population, Friedman’s test followed by Dunn’s post hoc test was used. In particular, this test was applied for the comparison of the immunoreactive areas of inflammatory cell subsets, and also for the comparison of their relative (per cent) portion in each neuritis population. For the analysis of PAR-1 and PAR-2 expression between NP, PCa and CP, and for the correlation of neural invasion with perineural mast cell infiltration, Kruskal-Wallis-Test in conjunction with Dunn’s post hoc test was used. For comparison of immunoreactivities of each inflammatory cell subtype, and of PAR-1 and PAR-2 neuro-immunoreactivities and expression levels between patients with pain and patients without pain, Mann-Whitney U Test was applied. For these two-group pain analyses, the unpaired t-test was additionally used to confirm the observed differences. For the mast cell subset analysis involving tryptase-chymase co-localization frequency, Fisher’s exact test was used. Results are expressed as median (Minimum; Maximum) except for the mRNA expression data which were presented as mean ± standard error of the mean (SEM). Two-sided p-values were always computed, and an effect was considered statistically significant at a *p*-value ≤ 0.05.

## Results

### Intrapancreatic nerves in PCa and CP are predominantly infiltrated by cytotoxic T lymphocytes, macrophages and mast cells

**Figure 1:**
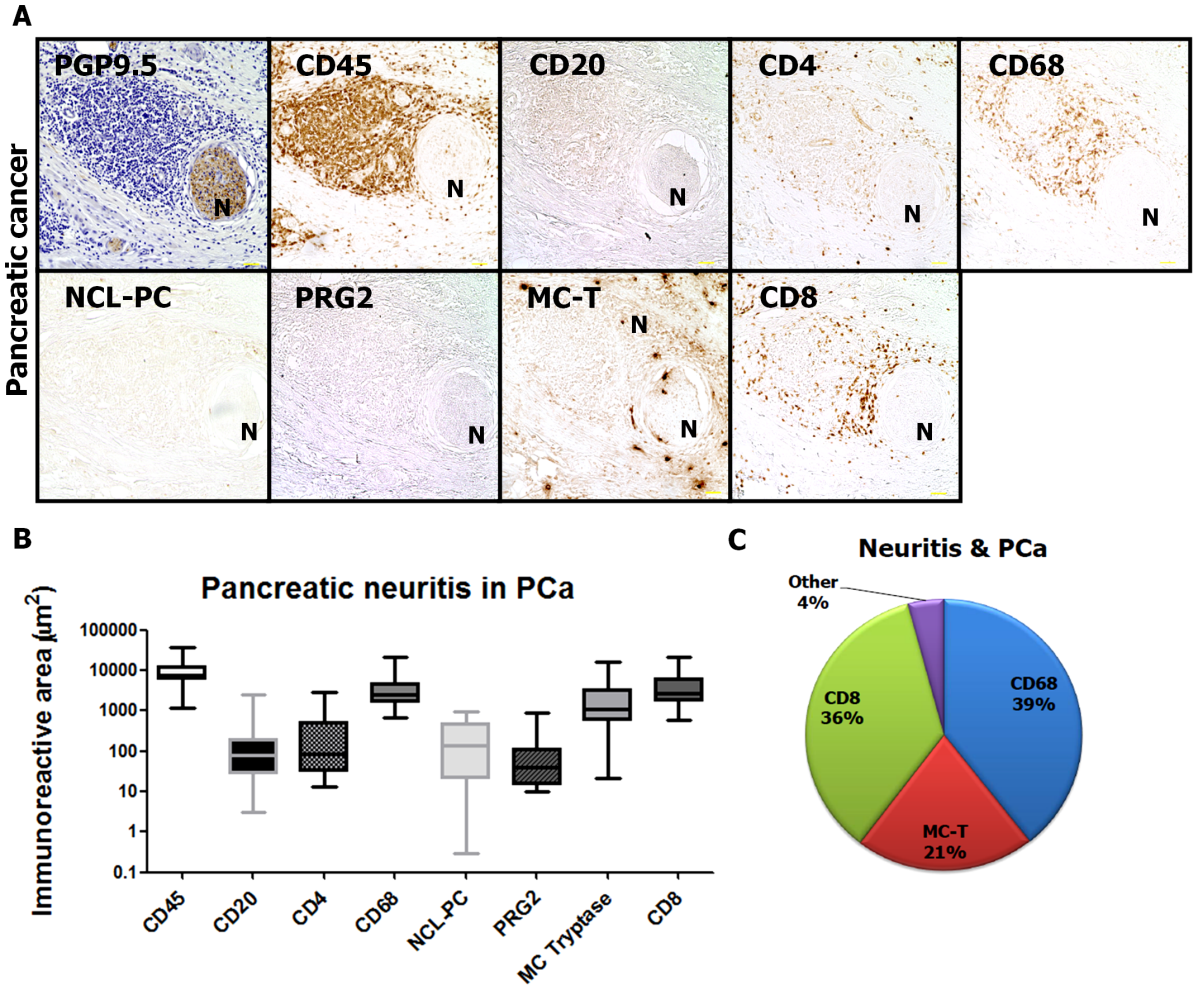
Inflammatory cell subsets composing pancreatic neuritis in pancreatic adenocarcinoma (PCa). (A) Pancreatic neuritis was identified by means of a hematoxylin-counterstained section which was immunolabeled with the pan-neuronal marker PGP9.5 or by means of a hematoyxlin-eosin (H&E)-stained section. Inflammatory cells composing pancreatic neuritis were identified by means of immunolabeling with the pan-leukocyte marker CD45, with CD68 (macrophages), CD8 (cytotoxic T-lymphocytes), CD4 (T-helper cells), CD20 (B-lymphocytes), NCL-PC (plasma cells), neutrophil elastase, PRG2 (eosinophils), anti-mast cell tryptase (MC-T) and correlated to pain sensation. (B–C) In PCa, CD68+ macrophages, CD8^+^ cytotoxic T lymphocytes and mast cells (MC) dominate the neural inflammatory cell population. “N” stands for the identified nerve. Between three to five nerves with pancreatic neuritis were analyzed from each patient. All images at 200x magnification. Scale bars indicate 50 µm.

As the main aim of the present study, we quantified the amount of leukocytes and leukocyte subpopulations in pancreatic neuritis. For this purpose, the area occupied by each leukocyte subtype was quantified via colorimetric analysis and expressed in µm^2^. In PCa, the pancreatic neuritis immune cell population was mainly composed of CD68^+^ macrophages [39.87% (10.15; 64.88) of all perineural inflammatory cells, 2545 µm^2^ (665.9; 22012)], followed by CD8^+^ cytotoxic T lymphocytes [33.20% (21.06; 54.11), 2623 µm^2^ (586.5; 21092)] and mast cells [MC, 14.46% (0.28; 52.10), 1106 µm^2^ (21.51; 16090), Figure 1A–C]. Importantly, all other major inflammatory cell populations were present in hardly detectable amounts [CD20^+^ B lymphocytes: 0.79% (0.10; 13.84) and 76.84 µm^2^ (3.06; 2598); CD4^+^ T helper cells: 1.61% (0.39; 28.45) and 83.03 µm^2^ (13.55; 2962); NCL-PC^+^ plasma cells: 1.87% (0.01; 10.54) and 140.9 µm^2^ (0.31; 986.9); PRG2^+^ eosinophils: 0.49% (0.10; 19.77) and 38.71 µm^2^ (9.93; 888.8); Figure 1A–C)]. The per cent distribution of leukocyte subpopulations in pancreatic neuritis in PCa is depicted in Figure 1C.

**Figure 2:**
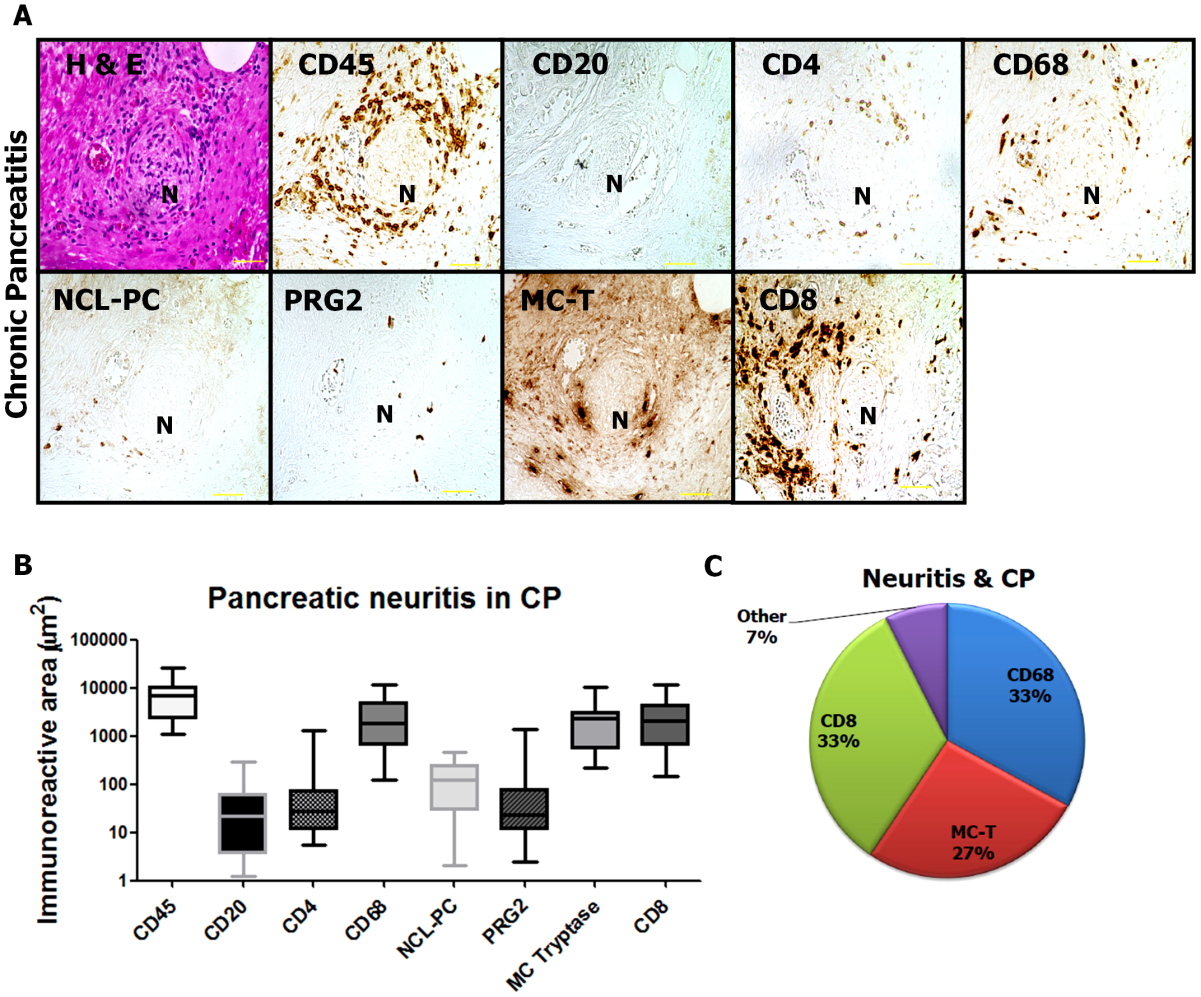
Inflammatory cell subsets composing pancreatic neuritis in chronic pancreatitis (CP). In chronic pancreatitis, pancreatic neuritis showed a similar composition to pancreatic cancer, comprising CD68+ macrophages, CD8^+^ cytotoxic T lymphocytes and mast cells (MC). “N” stands for the identified nerve. Between three to five nerves with pancreatic neuritis were analyzed from each patient. All images at 200x magnification. Scale bars indicate 50 µm.

In CP, the distribution of perineural inflammatory cell subtypes strongly resembled that in PCa. Also here, CD8^+^ cytotoxic T lymphocytes [34.17% (12.27; 78.97) and 2064 µm^2^ (142.7; 11334)], CD68^+^ macrophages [34.40% (1.562; 54.22) and 1852 µm^2^ (126.9; 11667)] and mast cells [24.16% (1.78; 56.63) and 2339 µm^2^ (221.1; 10155)] were most prevalent in pancreatic neuritis lesions (Figure 2A–C). The remaining inflammatory cell subtypes were only occasionally detected around intrapancreatic nerves [CD20^+^ B lymphocytes: 0.57% (0.07; 3.19) and 21.93 µm^2^ (1.27; 293.9); CD4^+^ T helper cells: 0.56% (0.16; 6.22) and 27.77 µm^2^ (5.38; 1325); NCL-PC^+^ plasma cells: 1.20% (0.05; 17.44)and 121.3 µm^2^ (2.08; 474.3); PRG2^+^ eosinophils: 0.61% (0.13; 6.24) and 23.33 µm^2^ (2.41; 1384); Figure 2A–C]. The per cent distribution of leukocyte subpopulations in pancreatic neuritis in CP is depicted in Figure 2C.

### Neuropathic pain in PCa is associated with specifically increased mast cell infiltration around intrapancreatic nerves

**Figure 3:**
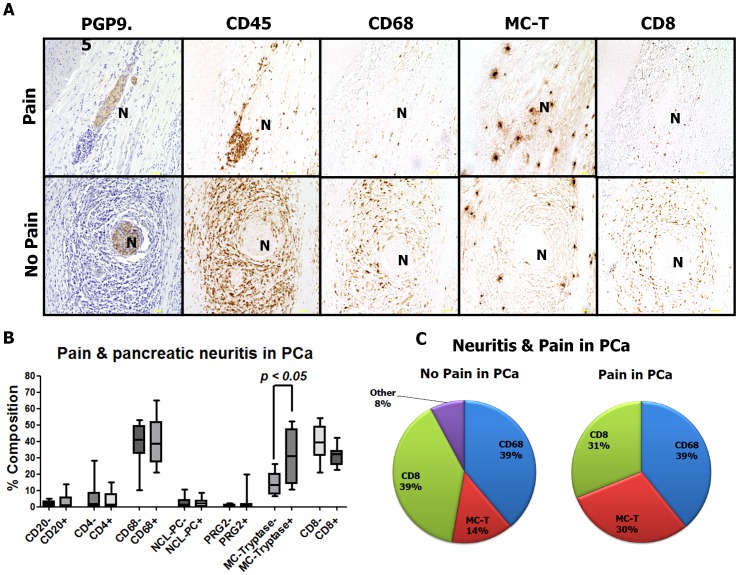
Impact of neuropathic pain upon the composition of pancreatic neuritis in pancreatic adenocarcinoma (PCa). Pain status of PCa patients did not affect the relative distribution of the majority of perineural inflammatory cell subsets in PCa, but it was only mast cells which were specifically enriched around intrapancreatic nerves of PCa patients with pain when compared to patients with no pain. “N” stands for the identified nerve. Between three to five nerves with pancreatic neuritis were analyzed from each patient. All images at 200x magnification. Scale bars indicate 50 µm.

One of the major aims of this study was to compare the distribution of the above-mentioned leukocyte subpopulations among PCa patients with and without abdominal pain. Looking at the whole amount of leukocytes in the neural inflammatory cell infiltrates, PCa patients with pain did not demonstrate any difference in the size of the inflammatory cell population per nerve [7574 µm^2^ (3121; 36358)] when compared to PCa patients without pain [7320 µm^2^ (1155; 33793)]. The analysis of inflammatory cell subtypes revealed that there was a shift in the distribution of only a single inflammatory cell subset among PCa patients with pain: The amount of perineural mast cells was specifically increased among PCa patients with pain [PCaP, 30.96% (10.75; 52.10) and 3081 µm^2^ (21.51; 16090)] than among patients without pain [PCaN, 13.41% (6.64; 26.29) and 777.6 µm^2^ (69.93; 3228), *p* < 0.05 Figure 3A–C]. All other inflammatory cell subtypes did not demonstrate any significant variation with the pain status of PCa patients [CD8^+^ cytotoxic T lymphocytes in PCaP: 32.22% (22.62; 42.07) and 2802 µm^2^ (116; 10933) vs. 39.57% (21.06; 54.11) and 2208 µm^2^ (586.5; 21092) in PCaN; CD68^+^ macrophages: 38.74% (21.21; 64.88) and 2628 µm^2^ (1214; 22012) in PCaP vs. 40.99% (10.15; 53.00) and 2486 µm^2^ (665.9; 8097) in PCaN; CD20^+^ B lymphocytes: 0.77% (0.23; 5.11) and 70.64 µm^2^ (3.36; 2598) in PCaP vs. 1.1% (0.10; 13.84) and 83.04 µm^2^ (3.06; 418.8) in PCaN; CD4^+^ T helper cells: 1.42% (0.39; 15.13) and 82.62 µm^2^ (30.62; 562.9) in PCaP vs. 1.79% (0.41; 28.45) and 83.03 µm^2^ (13.55; 2962) in PCaN; NCL-PC^+^ plasma cells: 2.08% (0.01; 8.61) and 245.4 µm^2^ (0.31; 986.9) in PCaP vs. 1.46% (0.20; 10.54) and 69.83 µm^2^ (9.50; 597.9) in PCaN; PRG2^+^ eosinophils: 0.50% (0.14; 19.77) and 48.98 µm^2^ (10.75; 888.8) in PCaP and 0.53% (0.10; 2.44) and 26.99 µm^2^ (9.93; 156.6) in PCaN; Figure 3A–C). The per cent distribution of leukocyte subpopulations in pancreatic neuritis among PCa patients with versus without pain is depicted in Figure 3C.

### Neuropathic pain in CP is characterized by increased perineural mast cell infiltration and suppression of perineural cytotoxic T lymphocytes in pancreatic neuritis

**Figure 4:**
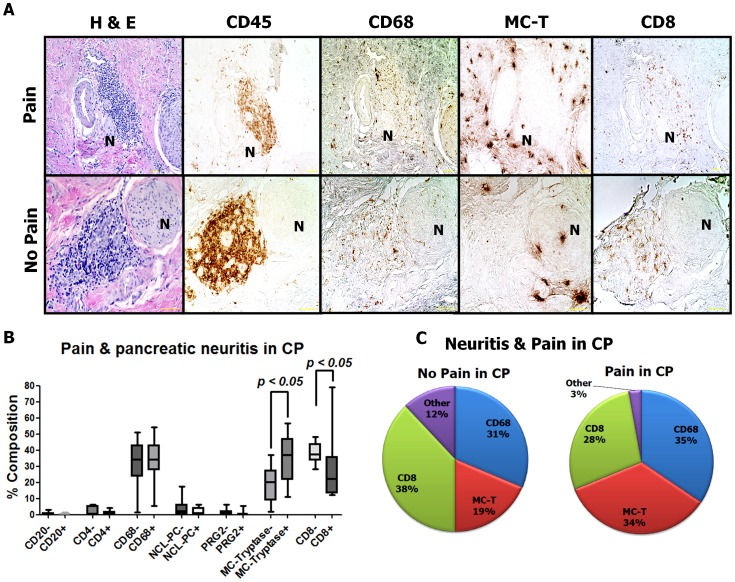
Impact of neuropathic pain upon the composition of pancreatic neuritis in chronic pancreatitis (CP). Similar to PCa, also in CP, patients with pain demonstrated a greater relative amount of mast cells (MC) around intrapancreatic nerves than patients without pain. Furthermore, this increase in the amount of perineural MC was paralleled by decreased amounts of cytotoxic CD8^+^ T lymphocytes around intrapancreatic nerves. “N” stands for the identified nerve. Between three to five nerves with pancreatic neuritis were analyzed from each patient. All images at 200x magnification. Scale bars indicate 50 µm.

Although pancreatic neuritis was first described in CP by some pioneering studies [5], [9], the influence of pain upon the distribution of inflammatory cell subsets in CP-associated pancreatic neuritis are widely unknown. When we compared the amount of leukocytes around intrapancreatic nerves in CP patients with vs. without pain, there were interestingly smaller amounts of perineural inflammatory cells among CP patients with pain [(CPP, median perineural CD45-immunoreactive area: 4333 µm^2^ (1118; 10375)] than among CP patients without pain [(CPN, median perineural CD45-immunoreactive area: 9831 µm^2^ (1687; 25685)]. This shrinkage of the perineural inflammatory cell infiltrate in CPP was accompanied by a specific increase in the relative proportion of mast cells in this inflammatory cell proportion [CPP: 36.93% (10.92; 56.63) vs. CPN: 20.25% (1.78; 37.22)], but not in the absolute amounts of perineural mast cells [CPP: 1546 µm^2^ (455.8; 3129) vs. CPN: 2833 µm^2^ (221.1; 7396)]. Furthermore, both the absolute and the relative amount of CD8^+^ T lymphocytes were diminished in painful CP [CPP: 22.35% (12.27; 78.97) and 768.6 µm^2^ (142.7; 11334) vs. CPN: 37.32% (28.31; 48.09) and 3628 µm^2^ (653.0; 7365)]. There were no further major alterations in the relative or absolute amounts of other inflammatory cell subtypes among CP patients with pain [CD68^+^ macrophages: 34.40% (5.57; 54.22) and 1409 µm^2^ (546.8; 4811) in CPP vs. 34.29% (1.56; 51.03) and 3129 µm^2^ (126.9; 6613) in CPN; CD20^+^ B lymphocytes: 0.50% (0.07; 1.65) and 7.07 µm^2^ (1.27; 40.86) in CPP vs. 0.76% (0.08; 3.19) and 61.42 µm^2^ (2.57; 293.9) in CPN; CD4^+^ T helper cells: 0.50% (0.16; 4.10) and 14.47 µm^2^ (5.38; 208.3) in CPP vs. 0.87% (0.28; 6.22) and 46.73 µm^2^ (21.97; 351.2) in CPN; NCL-PC^+^ plasma cells: 1.06% (0.05; 6.32) and 49.05 µm^2^ (2.08; 264.0) in CPP vs. 2.38% (0.45; 17.44) and 151.5 µm^2^ (8.58; 474.3) in CPN; PRG2^+^ eosinophils: 0.42% (0.15; 5.48) and 16.22 µm^2^ (2.41; 174.1) in CPP and 0.94% (0.13; 6.24) and 39.42 µm^2^ (3.34; 1384) in CPN; Figure 4A–C)]. The per cent distribution of leukocyte subpopulations in pancreatic neuritis among CP patients with versus without pain is depicted in Figure 4C.

### Perineural mast cell infiltration does not correlate to neural invasion in pancreatic cancer

**Figure 5:**
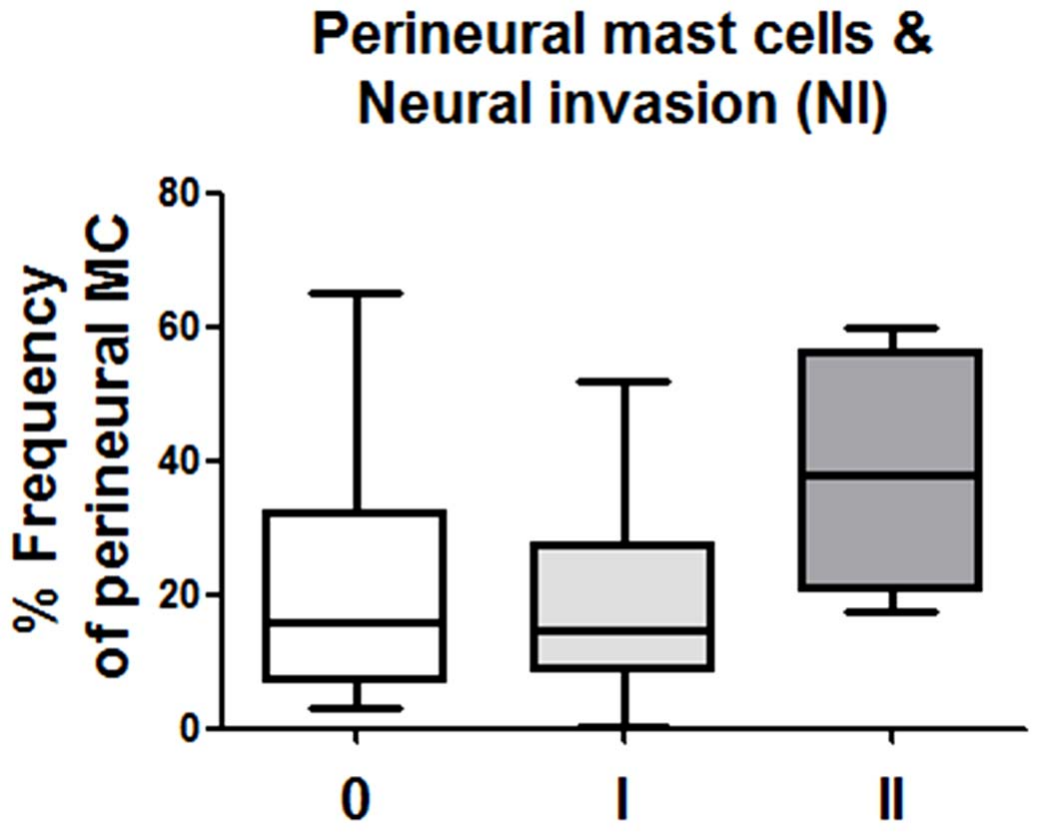
Neural invasion in pancreatic cancer (PCa) and perineural mast cell infiltration. Intrapancreatic nerves in PCa tissues were evaluated for the presence of neural invasion (NI) and classified into the NI degrees “0 / no invasion”, “1 / peri-neural invasion” and “2 / endo-neural invasion). In our cohort, the relative perineural mast cell amounts did not differ between the varying degrees of NI in human PCa. Results are presented as median (minimum; maximum).

Neural invasion (NI) is one of the histopathological hallmarks of PCa and is encountered in up to 100% of PCa specimens [12]. As we observed a specific increase in the amount of perineural MC in painful PCa, one major question which arose at this point was whether perineural MC infiltration also correlates to enhanced tumor invasion of nerves. For this purpose, we classified the severity of NI in PCa specimens into three categories (“0/no invasion”, “I/peri-neural invasion” and “II/endo-neural invasion”[1]) and correlated the NI severity to the relative amount of perineural MC. Here, there was no correlation between perineural MC infiltration and increasing severity of NI in PCa [no invasion/Grade 0: 15.73% (3.08; 65.16), peri-neural invasion/Grade I: 14.79% (0.34; 51.71) and endo-neural invasion/Grade II: 37.67% (17.65; 60.05), *p*  =  0.08, Figure 5].

### Expression and neural immunoreactivity of PAR-1 and PAR-2 in PCa and CP

**Figure 6:**
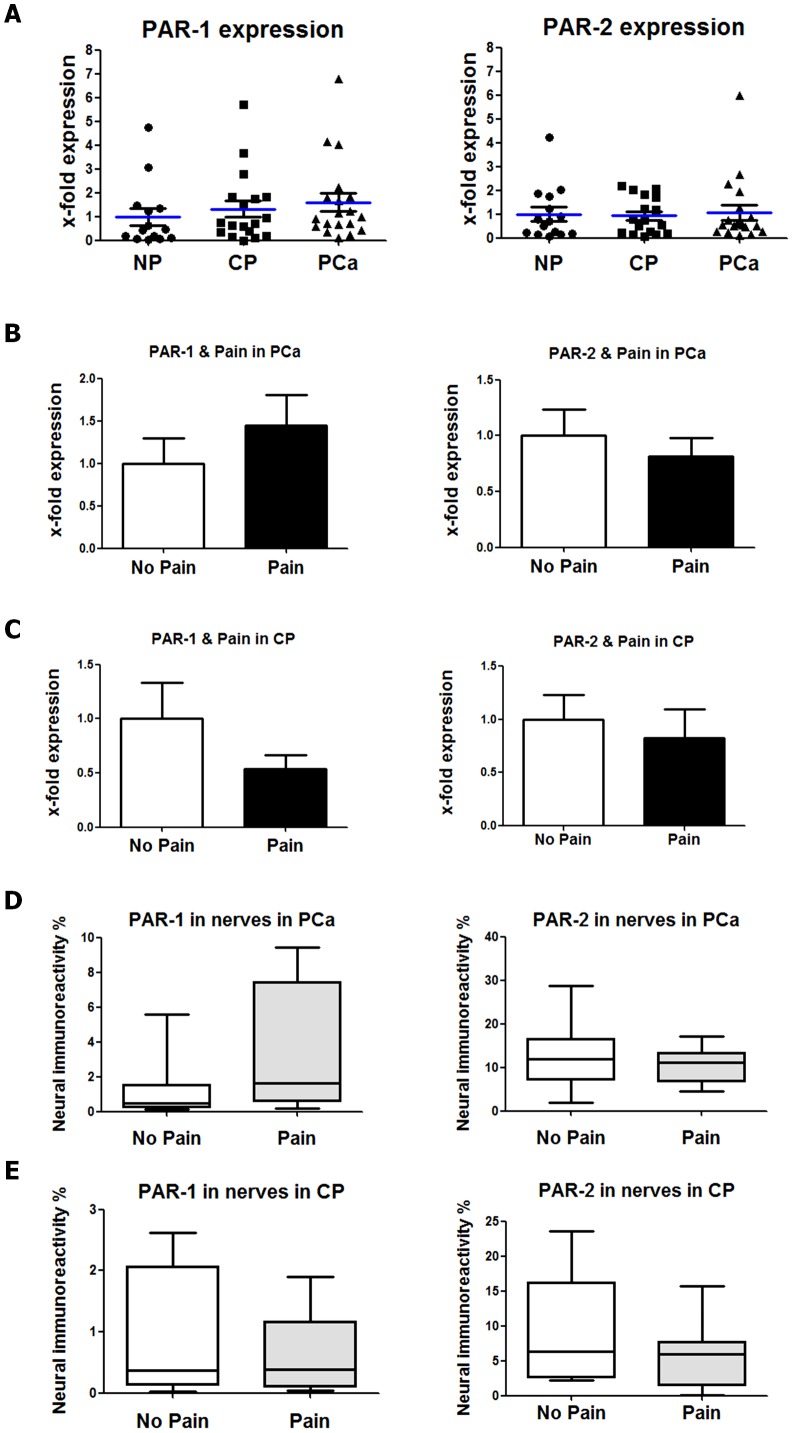
Protease-activated-receptor (PAR) type 1 (PAR-1) and type 2 (PAR-2) in human pancreatic cancer (PCa) and chronic pancreatitis (CP). (A) Expression of PAR-1 and PAR-2 was compared between normal human pancreas (NP), CP and PCa tissues via qRT-PCR and did not differ between these three entities. Expression was normalized first to the housekeeping gene cyclophilin B and then to NP. (B) In PCa, the tissue levels of PAR-1 and PAR-2 did not differ between patients with pain versus patients without pain. (C) Similarly, also in CP, there was no difference in the tissue mRNA levels of PAR-1 and PAR-2 in patients with no pain versus with pain. (D) Intrapancreatic nerves in PCa were analyzed for the immunoreactivity for PAR-1 and PAR-2 and correlated to the pain status of patients. Here, pain sensation was not associated with differences in the immunoreactivity of intrapancreatic nerves for PAR-1 or PAR-2. (E) In analogy with PCa, also in CP, patients with pain exhibited similar immunoreactivities in nerves for PAR-1 and PAR-2 as patients without pain.

Neurons can become sensitized by the action of thrombin upon the protease-activated receptor type 1 (PAR-1) or of MC-derived proteases (such as tryptase) upon protease-activated receptor type 2 (PAR-2) [13]. As we observed significantly elevated mast cell amounts around intrapancreatic nerves in PCa and CP, we also quantified the levels of PAR-1 and PAR-2 in these tissues and additionally in intrapancreatic nerves. Here, the comparison of mRNA expression for PAR-1 and PAR-2 in NP, PCa and CP did not show any major difference in the levels of these receptors (Figure 6A). Similarly, when PCa and CP patients were stratified into the “No Pain” and “Pain” groups, the mRNA levels of these receptors were not different between these groups (Figure 6B–C). When the neural immunoreactivities of these receptors were compared between patients with and without pain, PCa patients with pain tended to exhibit a slightly greater immunoreactivity for PAR-1 [3.272% (0.20;9.44)] than those without pain [1.22% (0.14;5.57), Figure 6D]. However, there was no difference in the PAR-2 immunoreactivity in PCa patients with pain [11.06% (4.45; 17.23)] versus without pain [11.90% (1.843; 28.64), (Figure 6D)]. Similarly, the pain status of CP patients had no influence on the neural immunoreactivity for PAR-1 or PAR-2 in our cohort (Figure 6E).

### Neuropathic pain-associated perineural mast cells in PCa and CP are MC_TC_–type mast cells

**Figure 7:**
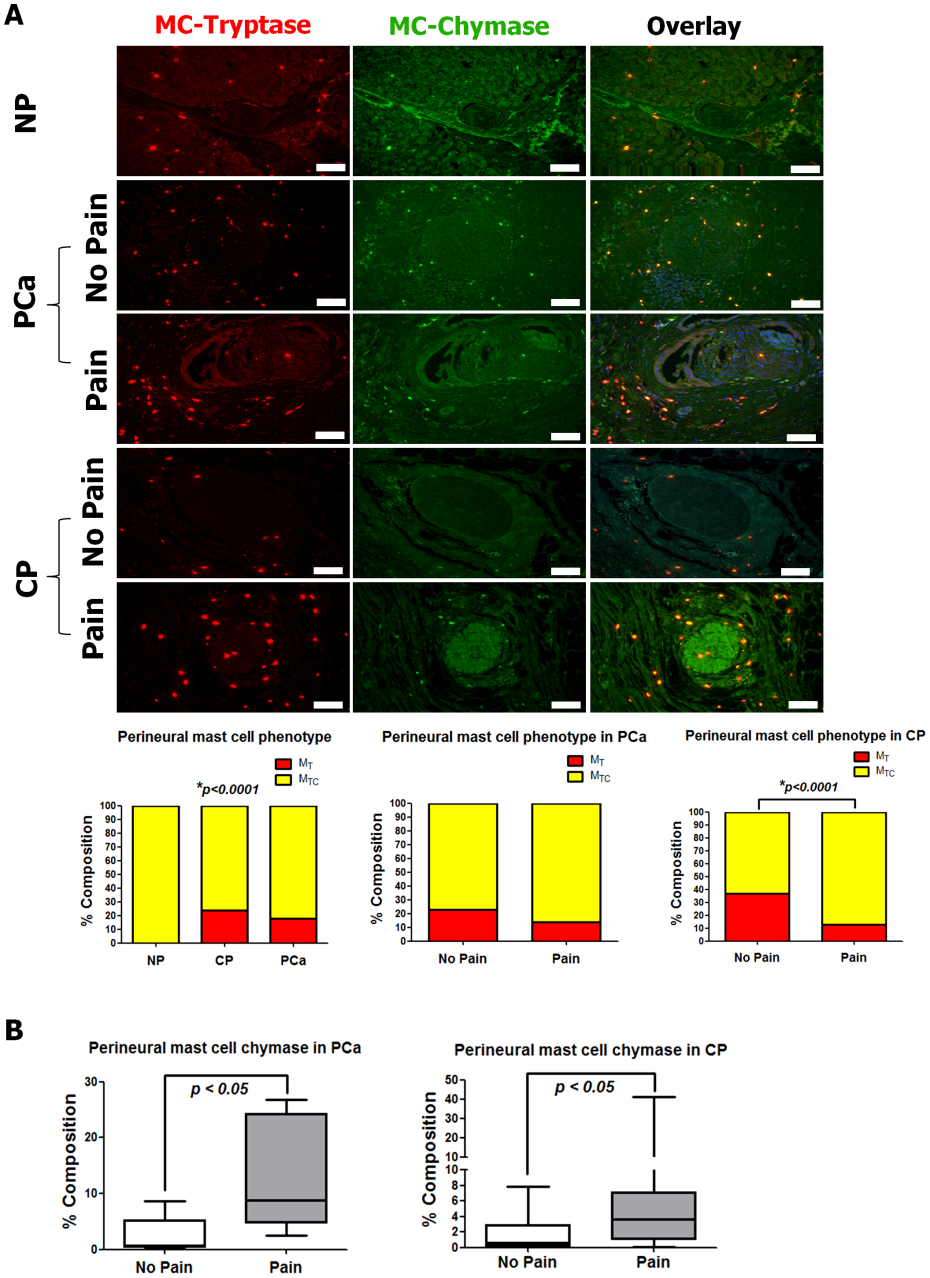
Analysis of mast cell (MC) phenotype in NP, PCa and CP. (A) Human pancreatic tissue samples from NP, PCa and CP were double-immunolabeled for perineural MC-tryptase (red) and MC-chymase (green). In all three entities, the vast majority of perineural MC demonstrated double immunoreactivity (yellow in overlay) for MC-tryptase and -chymase. In CP, there were significantly greater relative amounts of double-immunoreactive MC among patients with pain than among those without pain. The white scale bars indicate 100 µm. (B) In accordance with increased perineural MC-tryptase in painful PCa and CP, the perineural MC chymase immunoreactivity was significantly greater among PCa and CP patients with pain than in patients without pain. Results are expressed as median (Minimum; Maximum).

In humans, MC can be classified based on their mediator content into two categories: MC_T_-type mast cells which contain heparin and tryptase, and MC_TC_-type mast cells which additionally contain the proteolytic enzyme “chymase” [14]. To determine the prevalent mast cell phenotype in PCa, CP and NP, we performed double-immunofluoresence labelling in pancreatic tissues against mast cell tryptase and mast cell chymase. In NP, mast cells were nearly not detectable at all around intrapancreatic nerves. In all NP patients who were included in the analysis, we could only find three nerves which showed a close spatial association with inflammatory cells. The double immunolabeling of these mast cells showed that all of these cells contained both tryptase and chymase [100% (100;100)], thus classifying them as MC_TC_-type mast cells (Figure 7A). On the other hand, both in PCa and CP, MC_TC_ type mast cells were again the predominant perineural mast cell phenotype (81.7 ± 4.9% and 77.0 ± 4.0%, respectively), but both disease entities additionally contained perineural MC_T_ type (tryptase-only) mast cells (18.3 ± 4.9% and 23.7 ± 4.0%, respectively). The subgroup analysis considering the pain status of the patients showed a similar distribution of perineural MC_TC_ and MC_T_ type mast cells in PCa patients with pain (MC_TC_: 85.6 ± 6.4% and MC_T_: 14.4 ± 6.4%) versus those without pain (MC_TC_: 77.4 ± 7.4% and MC_T_: 22.6 ± 7.4%, Figure 6A). In contrast, CP patients with pain exhibited significantly greater proportions of perineural MC_TC_-type mast cells (MC_TC_: 86.9 ± 4.4% and MC_T_: 13.1 ± 4.3%, *p*<0.0001) than CP patients without pain (MC_TC_: 62.9 ± 6.4% and MC_T_: 37.1 ± 6.4%; Figure 6A).

Due this observed predominance of chymase-containing perineural mast cells in PCa and CP, we used mast cell chymase as additional marker to verify the specific increase of perineural mast cells in painful PCa and CP. Similar to our observations with mast cell tryptase labeling, pain in PCa and CP was associated with greater relative amounts of chymase-immunoreactive perineural mast cells [PCaP:8.73% (2.53; 16.82) and CPP: 3.70% (0.10; 41.26)] than pain-free PCa and CP [PCaN:0.75% (0.04; 8.71) and CPN: 0.65% (0.01; 7.87), *p*<0.05, Figure 6B].

## Discussion

The present study was designed to characterize the cellular subtypes within inflammatory cell infiltrates comprising pancreatic neuritis in human pancreatic cancer (PCa) and chronic pancreatitis (CP). It could demonstrate that cytotoxic T lymphocytes, macrophages and MC are the dominant cell populations in pancreatic neuritis in PCa and CP. Most importantly, out of all cells comprising pancreatic neuritis, it was only perineural MC which were uniquely increased in number around intrapancreatic nerves in PCa and CP patients with neuropathic abdominal pain sensation. The phenotype analysis of these MC revealed their predominant phenotype as chymase-containing MC_TC_ cells.

The presence of pancreatic neuritis is known to correlate to the severity of abdominal pain sensation of PCa and CP patients [1], [5]. Furthermore, its degree also bears an association with neuroplastic alterations in PCa and CP, including increased neural density and neural hypertrophy [1]. Moreover, pancreatic neuritis represents the pancreatic counterpart of visceral neuro-inflammation, which is encountered in numerous gastrointestinal (GI) disorders including inflammatory bowel disease (IBD) [15], irritable bowel syndrome (IBS) [16] and appendicitis [17]. In all these disorders, visceral neuro-inflammation is closely related to the generation of pain and organ dysfunction [15]. Therefore, the implications of our findings could be understood in the light of these important aspects of visceral neuro-inflammation.

The generation of pancreatic pain is still only incompletely understood [18], [19]. So far, it has become clear that sensitization of nociceptors and neuroplastic alterations at the periphery (i.e. in the pancreas) are closely related to the severity of pain of PCa and CP patients [20]. For CP, it has further been shown that concomitant plasticity in viscero-sensory areas of the central nervous system is present among CP patients with severe pain sensation [19]. The major question which arises at this point is whether perineural intrapancreatic MC can be responsible for the induction of peripheral nociceptive sensitization and peripheral neuroplasticity with consequent central hypersensitivity.

Indeed, there is a considerable number of visceral painful disorders in which MC have been shown to be specifically enriched around intra-organ nerve fibers and correlate to the extent of pain sensation [21]. MC are resident immune cells in the GI tract, skin, lung, brain and other tissues and are classically known as mediators of type I hypersensitivity reactions to allergens or anaphylactic agents by releasing a large set of MC-derived degranulation products like histamine, serotonin, cytokines, prostaglandins, etc. [22]. Moreover, they exhibit a very close spatial association with intra-organ nerves (found sometimes as close as 20 nm away from nerve endings[23]) and a bi-directional mast-cell-neuron-communication [22]. Particularly, mast cells are found in close association with peptidergic nerve fibers containing substance P (SP), calcitonin-gene-related-peptide (CGRP) [24] or those containing nerve growth factor (NGF) which can all bind to their specific receptors on MC and entail MC activation and degranulation [25]–[27]. Conversely, activated degranulating MC release neuron-activating molecules like histamine, serotonin, NGF, proteases including MC tryptase which can also activate and sensitize peripheral neurons via their corresponding receptors (H1-4, 5HT-3, tyrosine-kinase-receptor A/TrkA, protease-activated-receptor-1/PAR-1) causing pain and dysregulation of neuronal function [28]. Accordingly, neuron-MC-interactions are intensively investigated to understand the pathogenesis of pain in several disorders including migraine, interstitial cystitis, ulcerative colitis, etc.[16], [29], [30]. In IBS, administration of the MC stabilizing agent ketotifen was recently shown to relieve abdominal pain of IBS patients [31]. Ketotifen is a MC-stabilizing and antihistaminic agent and can inhibit the release of neuron-activating MC-mediators like histamine and tryptase from MC [32]. Based on our results and previous observations on IBS, we believe that administration of such MC-stabilizing agents may reduce the action of these neuron-activating MC-derived factors and can have similar analgesic effects in human PCa and CP.

Neuroplasticity in PCa and CP is characterized by increased presence of neurotrophic factors like NGF, artemin, neurturin and their receptors in intrapancreatic nerves [33], [34]. Furthermore, in CP, increased expression of the neuropeptides SP and CGRP has been reported to correlate to the pain sensation of CP patients [35]. Anti-NGF-antibody administration was shown to decrease pancreatic nociceptor excitability in a rat model of CP [36]. Furthermore, in the course of neurogenic inflammation, protease-activated receptors (PAR) are crucially involved in the generation of pancreatic pain [37], [38]. Therefore, current literature contains a large series of studies which could demonstrate the overexpression and the involvement of numerous key mediators in the generation of pancreatic pain, and all these molecules are actually known to be crucially involved in the bi-directional communication between MC and nociceptive neurons. Based on our analysis of PAR-1 and PAR-2 in PCa and CP, it seems that these receptors are not differentially regulated in these diseases. However, their presence in intrapancreatic nerves suggests that they may still serve as the receptors for MC-derived proteases.

Intratumoral MC infiltration is a well characterized factor promoting PCa cell invasiveness. Indeed, MC-conditioned media were previously reported to increase the invasiveness, proliferation and migration of PCa cells in vitro [39]. Furthermore, MC infiltration was reported to correlate to higher tumor grade and diminished survival in PCa [39]. However, in our study, there was no correlation between perineural MC infiltration and increasing severity of neural invasion in PCa. Therefore, looking at our results, we assume that perineural mast cell infiltration is primarily related to neuropathic pain and less to the extent of tumor invasion in PCa.

Interestingly, there was a single study which ascribed a role to MC in pancreatic nociception [40]. Here, Hoogerwerf et al. reported increased MC counts in the *whole* pancreatic tissue of CP patients and could also demonstrate decreased abdominal tactile sensitivity of MC-deficient mice with CP [40]. However, the study did not investigate the role of perineural MC which are known to be the actual interacting partner for neurons [23]. Furthermore, there are no other studies in the literature which addressed the potential role of MC in pancreatic neuropathic pain in human PCa.

In summary, the present study elucidated for the first time the major subtypes of inflammatory cells involved in pancreatic neuritis in PCa and CP. Pancreatic neuritis lesions in PCa and CP are mainly composed of cytotoxic T-lymphocytes, macrophages and MC. However, in both disease entities, it is only MC which are uniquely increased in number around intrapancreatic nerves of patients with concomitant abdominal neuropathic pain sensation.

In conclusion, due to the well-established role of MC in numerous painful disorders including enteric neuropathies, MC may be the key inflammatory cell subtype in the generation of pancreatic nociceptor hyperexcitabilty in PCa and CP. Therefore, future studies shall investigate the impact of MC-modulation or MC-stabilization upon pain sensation in experimental and clinical PCa and CP.
